# COVID-19 Shows That Health Education Programs in Iran Must Be Revised

**DOI:** 10.1177/1010539520947895

**Published:** 2020-08-07

**Authors:** Hamid Jafari, Majid Heidari Jamebozorgi, Majid Amiri Gharaghani

**Affiliations:** 1 Sirjan School of Medical Sciences, Sirjan, Iran

The outbreak of new coronavirus disease 2019 (COVID-19) in China in December 2019 and then globally is of most concern for public health authorities worldwide. The novel coronavirus has been declared the sixth public health emergency of international concern by the World Health Organization and subsequently named COVID-19.^1,2^ As of June 1, 2020, more than 6.2 million confirmed cases and about 374 000 deaths have been documented across the world. In Iran, to this date 151 000 cases were confirmed and 7797 people died from COVID-19.^3^

As the virus spread across the country, the government and the Ministry of Health and Medical Education pursued a policy of social distancing. People’s gathering places, including religious holy places, sports gyms, cinemas and theaters, swimming pools and entertainment complexes, shopping malls, and other public places, were closed.^4^ People were expected to refrain from engaging in risky behaviors with the created atmosphere, but some unsafe behaviors that show a lack of awareness about health issues were prevalent in society. The most important unsafe behaviors are ignorance of instructions for visiting religious places, eating denatured alcohol (methanol) that caused the death of more than 500 people,^5^ gathering in entertainment venues and going on trips, not using protective equipment in public places such as masks, and reference to the use of some nonhygienic substances, on the advice of nonspecialists, such as opium, feces, and so on.

Why such behaviors are so prevalent in a country like Iran with a brilliant track record in primary health care needs to be investigated. The prevalence of such high-risk behaviors indicates that health education has not been well implemented and educational programs and policies to increase health literacy in the country need to be reviewed.^6^ Given that a small percentage of people in Iran have a health-related education,^7^ it is necessary to teach students in the early stages of school to follow healthy behaviors. Community participation through social leaders and celebrities can also be used as a good educational policy.

In this situation, the first and most important strategy is to focus on society’s culture. Increasing educational activities for people about correct individual and social behaviors, and working on changing people’s attitudes in order to accept the social distance plan can be very effective and useful. Correct and principled policies by the authorities, providing livelihood support to low-income people, paying attention to vulnerable groups such as patients with immunodeficiency or patients with underlying disease, controlling rumors, controlling anxiety and stress management, strengthening popular dynamics, strengthening participation of people and the use of people’s capacities, expanding physical strengthening skills with home sports and recreation at home, providing water and electricity savings strategies, and so on, at home, and finally applying punitive measures can be effective and useful.
